# A single-domain i-body, AD-114, attenuates renal fibrosis through blockade of CXCR4

**DOI:** 10.1172/jci.insight.143018

**Published:** 2022-02-22

**Authors:** Qinghua Cao, Chunling Huang, Hao Yi, Anthony J. Gill, Angela Chou, Michael Foley, Chris G. Hosking, Kevin K. Lim, Cristina F. Triffon, Ying Shi, Xin-Ming Chen, Carol A. Pollock

**Affiliations:** 1 Renal Medicine, Kolling Institute of Medical Research, Sydney Medical School, University of Sydney, Sydney, New South Wales, Australia.; 2 Department of Anatomical Pathology, NSW Health Pathology, Royal North Shore Hospital, Sydney, New South Wales, Australia.; 3 The Department of Biochemistry and Chemistry, La Trobe Institute for Molecular Science, La Trobe University, Melbourne, Victoria, Australia.; 4 AdAlta Limited, Melbourne, Victoria, Australia.

**Keywords:** Nephrology, Therapeutics, Chemokines, Chronic kidney disease, Fibrosis

## Abstract

The G protein–coupled CXC chemokine receptor 4 (CXCR4) is a candidate therapeutic target for tissue fibrosis. A fully human single-domain antibody-like scaffold i-body AD-114-PA600 (AD-114) with specific high binding affinity to CXCR4 has been developed. To define its renoprotective role, AD-114 was administrated in a mouse model of renal fibrosis induced by folic acid (FA). Increased extracellular matrix (ECM) accumulation, macrophage infiltration, inflammatory response, TGF-β1 expression, and fibroblast activation were observed in kidneys of mice with FA-induced nephropathy. These markers were normalized or partially reversed by AD-114 treatment. In vitro studies demonstrated AD-114 blocked TGF-β1–induced upregulated expression of ECM, matrix metalloproteinase-2, and downstream p38 mitogen-activated protein kinase (p38 MAPK) and PI3K/AKT/mTOR signaling pathways in a renal proximal tubular cell line. Additionally, these renoprotective effects were validated in a second model of unilateral ureteral obstruction using a second generation of AD-114 (Fc-fused AD-114, also named AD-214). Collectively, these results suggest a renoprotective role of AD-114 as it inhibited the chemotactic function of CXCR4 as well as blocked CXCR4 downstream p38 MAPK and PI3K/AKT/mTOR signaling, which establish a therapeutic strategy for AD-114 targeting CXCR4 to limit renal fibrosis.

## Introduction

Fibrosis is a pathologic variant of the normal wound healing process characterized by injury, inflammation, myofibroblast activation, and matrix accumulation and remodeling (1). Fibrotic diseases have been estimated to account for up to half of deaths in the developed world (2). However, there is no clinically satisfactory therapeutic approach to fibrosis. Chronic kidney disease (CKD) is an epidemic increasing at an alarming rate (3). Kidney fibrosis is a major contributor to progression of almost all forms of CKD (4). Despite many attempts to block kidney fibrosis, few have been approved in clinical practice and a large treatment gap remains. Thus, novel antifibrotic therapies are urgently needed to prevent the progression of kidney fibrosis in CKD.

Proximal tubular cells (PTCs), which comprise the bulk of renal parenchyma, are the primary target of various ischemic, metabolic, immunologic, and toxic insults (5). In many animal models of CKD, such as folic acid (FA) nephropathy and unilateral ureteral obstruction (UUO), tubular injury has been observed (6). During the induction phase of chronic kidney injury, PTCs actively participate in injurious pathways predominantly through their ability to synthesize reactive oxygen species and inflammatory mediators, including chemokines (5). Chemokine receptor CXCR4 is a member of the CXC chemokine receptor family of G protein–coupled receptors (7). In human biopsies of IgA nephropathy, minimal change nephrotic syndrome, focal segmental glomerulosclerosis, chronic pyelonephritis, and acute tubular necrosis, there is intense CXCR4 staining in proximal tubules (8). Importantly, CXCR4 has been demonstrated to play a central role in the development of other fibrotic diseases, such as nonalcoholic steatohepatitis (9), ocular neovascularization (10), hypertrophic scar formation (11), and diabetic cardiac fibrosis (12). In the UUO nephropathy mouse model, CXCR4 is significantly upregulated, and the nonpeptide CXCR4 antagonist AMD3100 blunts the UUO-induced fibrotic response (13). Additional CXCR4 inhibitors include the small molecule MSX-122 (14), peptides LY2510924 (15) and BL-8040 (16, 17), and antibodies such as BMS-936564 and PF-06747143 (18–20). However, the only CXCR4 antagonist approved by the US FDA has been AMD3100, the clinical trial of which was terminated due to its poor oral bioavailability and its off-target cardiotoxicity (21).

The i-body AD-114-PA600 (AD-114), a fully humanized single-domain antibody-like scaffold with specific high binding affinity to CXCR4, was designed using information from the IgNAR antibodies from sharks (22). AD-114 has been reported to inhibit idiopathic pulmonary fibrosis in in vitro and in animal studies through different mechanisms compared with other CXCR4 antagonists (23). Briefly, AD-114 can penetrate deep into the ligand binding pocket, contact residues that were previously only accessible to small molecule drugs, and selectively block CXCR4 signaling (22). Moreover, AD-114 can block inflammatory cell migration without stem cell mobilization (22). In this study, we have evaluated the renoprotective role of AD-114 in FA nephropathy, a well-characterized and widely used animal model of renal interstitial fibrosis, and examined the effects of AD-114 on TGF-β1–induced fibrotic responses in renal PTCs in vitro. These results were then confirmed in an alternative model of renal fibrosis, i.e., the UUO model using AD-214. I-body AD-214 consists of AD-114 with a human Fc fragment fused at the C-terminus of the i-body. Taken together, our results suggest that targeting CXCR4 using the i-body AD-114 or the second generation AD-214 is a novel and effective approach for both the prevention and treatment of fibrotic CKD.

## Results

### CXCR4 expression is upregulated in mouse and human fibrotic kidneys.

**Figure 1:**
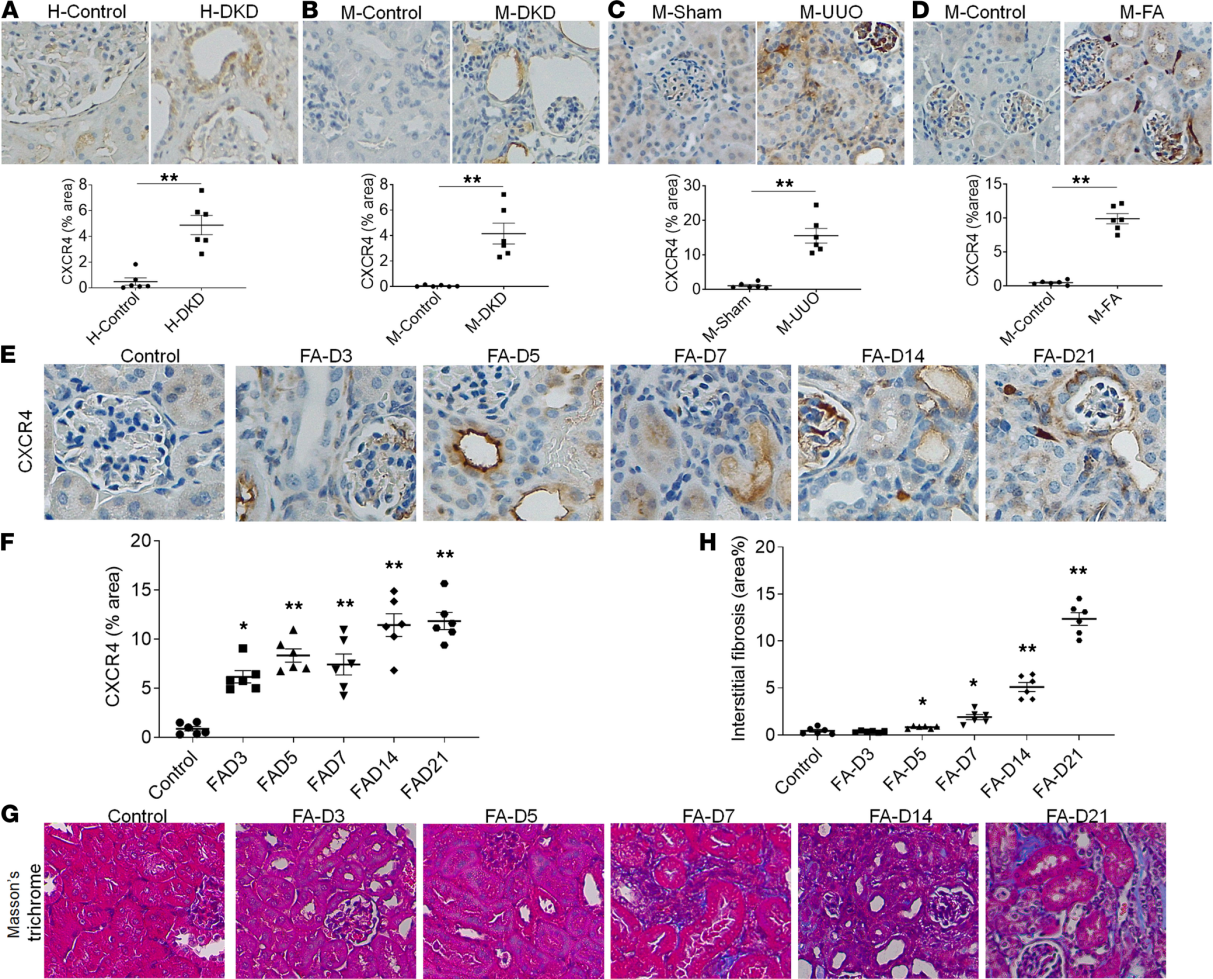
FA induces significant upregulation of CXCR4 and kidney interstitial fibrosis. IHC staining and quantitation of CXCR4 expression in fibrotic kidneys from (**A**) patients with DKD, (**B**) STZ–endothelial nitric oxide synthase–deficient (STZ-eNOS^–/–^) DKD mice, (**C**) UUO mice, and (**D**) FA nephropathy mice. H, human; M, mouse. *n* = 6. Data are shown as mean ± SEM and were analyzed by Student’s unpaired 2-tailed *t* test. (**E** and **F**) IHC staining and quantitation of CXCR4 levels in mouse kidneys at days 3, 5, 7, 14, and 21 after FA injection in mice. (**G**) Representative images of Masson’s trichrome staining of kidneys at days 3, 5, 7, 14, and 21 after treatment with FA. (**H**) The degree of tubulointerstitial fibrosis was determined by ImageJ software (NIH). Original magnification: ×400 (**E**), ×200 (**A**–**D** and **G**). Statistical analysis was performed using 1-way ANOVA followed by Tukey’s multiple comparisons test. Results are presented as mean ± SEM. *n* = 6. *P < 0.05, **P < 0.01.

Although CXCR4 is highly expressed in the embryonic kidney, its expression is significantly lower in healthy adult kidneys (8, 24). To detect CXCR4 expression in fibrotic kidneys, kidney biopsies from normal nephrectomy specimens and patients with diabetic kidney disease (DKD) and kidney fibrosis and kidneys from 3 mechanistically distinct mouse models of kidney fibrosis, including streptozotocin-induced (STZ-induced) DKD, UUO, and FA nephropathy, were collected. CXCR4 expression was detected using immunohistochemistry (IHC). As shown in Figure 1, A and B, CXCR4 expression was significantly upregulated in diabetic kidneys from patients and eNOS^–/–^ DKD mice compared with nondiabetic control (*P* < 0.01). Compared with control, day 14 UUO kidneys and day 21 FA kidneys had an approximately 15-fold and approximately 20-fold increase, respectively, in CXCR4 expression, which was predominantly localized in renal tubular cells (Figure 1, C and D, *P* < 0.01). Collectively, these data indicated that CXCR4 expression is upregulated significantly in humans with DKD and in 3 fibrotic mouse models, of which FA nephropathy had the highest increase in CXCR4 expression. Thus, the FA nephropathy model was used for the following in vivo study.

To assess CXCR4 expression at early stages of FA nephropathy, mice were culled at different time points (days 3, 5, 7, 14, 21) after treatment with FA (animal study 1). As shown in Figure 1, E and F, a significant increase of CXCR4 was observed from day 3 after FA injection, and CXCR4 increased approximately 9-fold at day 5, with a subsequent slight decrease at day 7. This upregulation of CXCR4 at an early stage may lay the foundation for a preventative effect of AD-114 on subsequent renal fibrosis. CXCR4 expression was predominantly localized to renal tubular cells. This is consistent with previous literature that tubular and infiltrating immune cells contribute to the increased CXCR4 content observed in diseased kidneys due to varying etiologies.

To evaluate the degree of progressive kidney fibrosis after FA administration, kidneys harvested at different time points were also analyzed by Masson’s trichrome staining and IHC. The Masson’s trichrome–stained kidney displayed marked interstitial fibrosis on visual inspection from day 7 (Figure 1G). Quantitative analysis of tubulointerstitial fibrosis indicated that FA induced significant fibrosis compared with control from day 5 as shown by the bar graph in Figure 1H. Consistently, a marked induction of fibrotic markers collagen-1 (COL-1), collagen-3 (COL-3), and fibronectin (FN) was observed at day 7 (COL-1: 6.2-fold, COL-3: 5.8-fold, FN: 25.5-fold, *P* < 0.05, Supplemental Figure 1; supplemental material available online with this article; https://doi.org/10.1172/jci.insight.143018DS1).

### Co-incident administration of i-body AD-114 ameliorates FA-induced kidney fibrosis.

**Figure 2:**
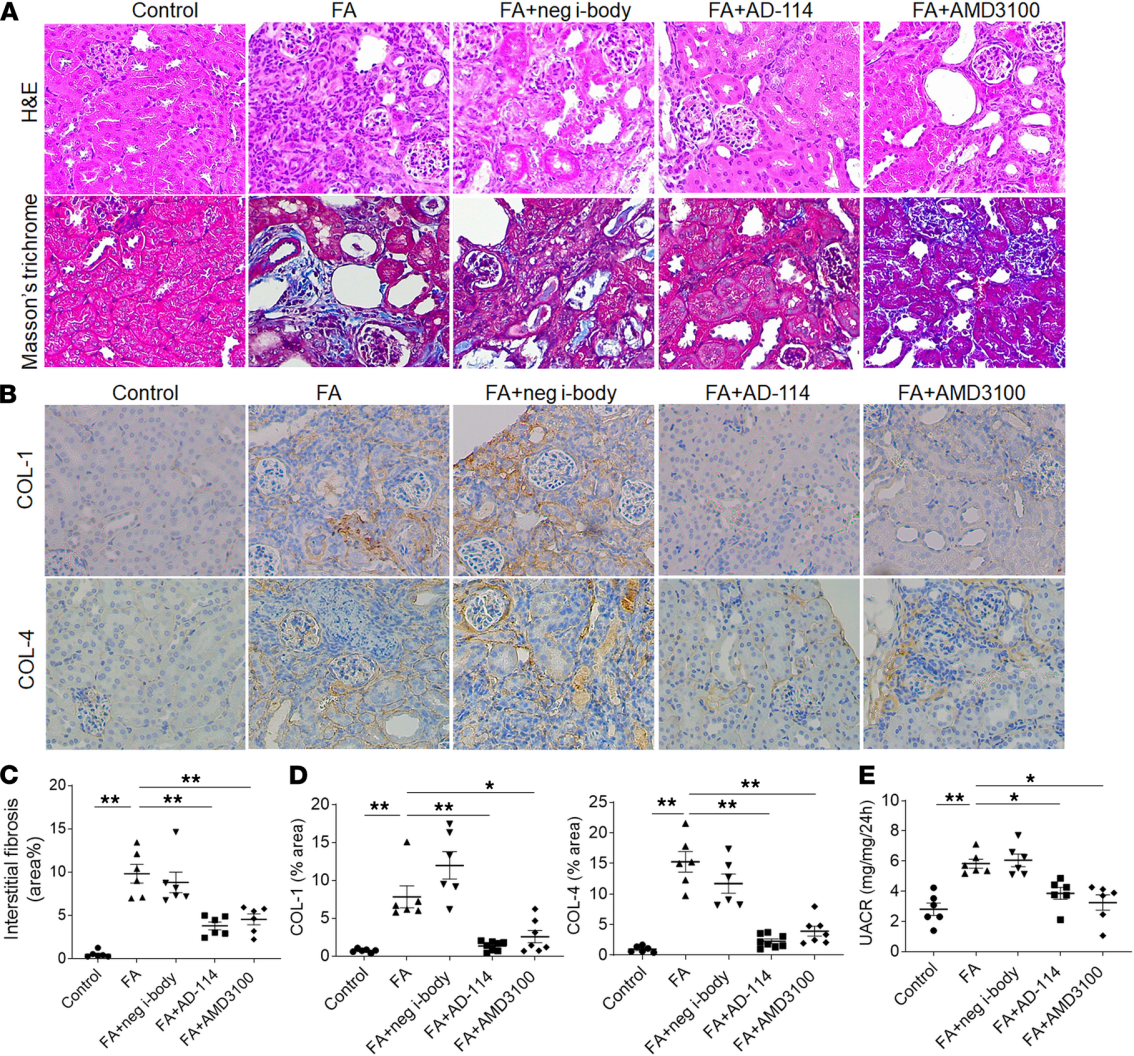
I-body AD-114 ameliorates FA-induced renal fibrotic responses and improves kidney function in the preventative mouse model. Mice were dosed 1 hour prior to FA with negative i-body, AD-114, or AMD3100 i.p. and were then administrated daily with these treatments until day 21 in animal study 2. (**A**) Representative images of H&E and Masson’s trichrome staining. (**B**) Representative images of COL-1– and COL-4–stained kidney sections. (**C**) Quantitative analysis of tubulointerstitial fibrosis from Masson’s trichrome staining by ImageJ software. (**D**) Quantitation of COL-1 and COL-4 immunohistochemical staining. (**E**) A 24-hour urine was collected at day 21, and urinary albumin and creatinine levels were detected for UACR calculation. Original magnification: ×200 in all. Statistical analysis was performed using 1-way ANOVA followed by Tukey’s multiple comparisons test. Results are presented as mean ± SEM. *P < 0.05, **P < 0.01. *n* = 6–8.

As i-body AD-114 has been reported to block CXCR4 signaling pathways (22), we sought to test its efficacy in mitigating renal fibrosis in vivo. In the preventative study, mice were dosed i.p. 1 hour prior to FA injection with negative control i-body 21H5-Im7-FH (21H5), AD-114, or AMD3100 (serving as positive control) and were then dosed daily with these treatments until day 21 (Supplemental Figure 2). As demonstrated by H&E and Masson’s trichrome staining in Figure 2A, the kidney displayed typical features of renal fibrosis in the FA and FA+negative i-body groups. In contrast, the severity of tubulointerstitial fibrosis was dramatically decreased by co-incident administration of AD-114 (62% reduction) relative to FA alone (Figure 2, A and C, *P* < 0.01).

Consistent with the Masson’s trichrome staining data, IHC staining revealed that FA-induced deposition of fibrotic markers COL-1 and COL-4 was significantly attenuated, by 82% for COL-1 and 84% for COL-4 (Figure 2, B and D, *P* < 0.01), with AD-114 treatment. These data indicated that the i-body AD-114 can suppress ECM overproduction and reduce renal fibrosis in the mouse model of FA nephropathy. Stromal cell-derived factor-1 (SDF-1, also known as CXCL12) is a CXC chemokine and the principal ligand for CXCR4. As shown in Supplemental Figure 3, A and B, we observed a significant increase of SDF-1 in kidneys after FA injection. However, this increased level of SDF-1 was not inhibited by administration of AD-114 (*P* > 0.05), indicating that AD-114 had no effect on the expression of SDF-1.

To assess whether reduced kidney fibrosis was associated with improved physiologic parameters, a 24-hour urine was collected at the time of sacrifice, and the urinary albumin/creatinine ratio (UACR) was measured. It was shown that albuminuria developed in the FA mice, which was reduced by 38% in mice treated with AD-114 (Figure 2E, *P* < 0.05).

### I-body AD-114 inhibits inflammatory responses as well as macrophage infiltration and activation in FA-induced kidney fibrosis.

Chronic inflammation plays an essential role in the progression of CKD. During inflammation, leukocytes are recruited to the glomerulus and renal interstitium, leading to increased secretion of chemokines or chemotactic cytokines (25). The gradient of chemokines further drives infiltration of monocytes/macrophages, DCs, and T and B cells to the injured site and production of inflammatory cytokines, such as TNF-α and IL-6, and growth factor TGF-β1 (26). The chemokine receptor CXCR4 is involved in inflammatory cytokine production and exhibits substantial chemoattractive activity for various inflammatory cells. Among all the cell types, the macrophage is the cell type in most models of CKD that is highly associated with tubulointerstitial fibrosis and poor renal outcomes (27–29).

**Figure 3:**
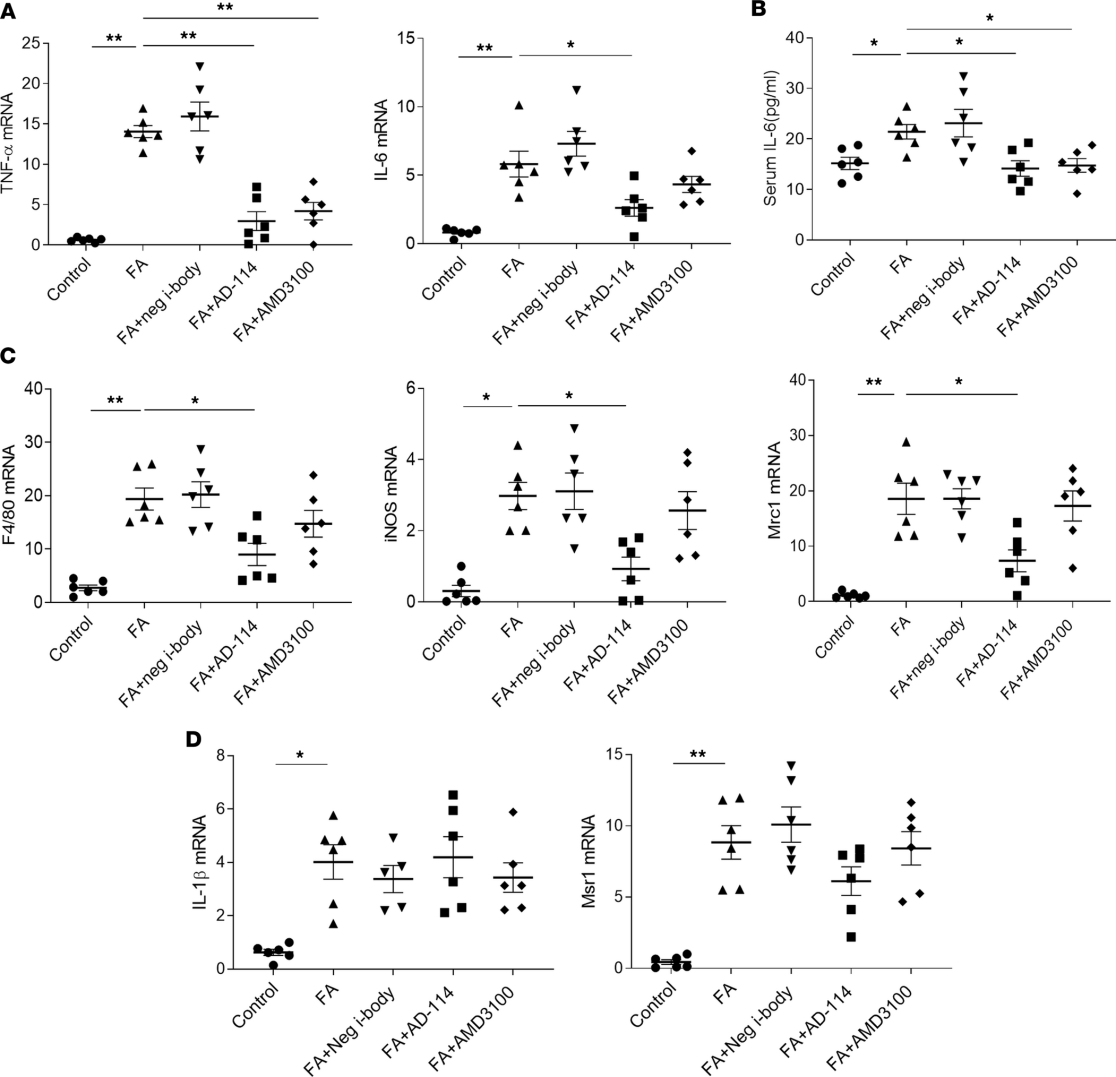
I-body AD-114 inhibits inflammatory responses as well as macrophage infiltration and activation in FA-induced kidney fibrosis. (**A**) mRNA expression of TNF-α and IL-6 in mouse kidneys was measured by quantitative real-time PCR (RT-PCR). (**B**) Serum IL-6 levels were detected by ELISA. (**C** and **D**) mRNA expression of F4/80, iNOS, Mrc1, IL-1β, and Msr1 in mouse kidneys was measured by quantitative RT-PCR. β-Actin was used as the endogenous control gene. Statistical analysis was performed using 1-way ANOVA followed by Tukey’s multiple comparisons test. Results are presented as mean ± SEM. *P < 0.05, **P < 0.01. *n* = 6.

It has been reported that i-body AD-114 specifically antagonizes CXCR4, disrupts CXCR4/SDF-1 interactions, and blocks leukocyte recruitment and inflammatory cell migration but was unable to mobilize stem cells in the assays used (22). To characterize the role of AD-114 in the regulation of inflammation in FA nephropathy, 2 inflammatory cytokines, TNF-α and IL-6, were examined in kidney tissue. It was demonstrated that the expression of TNF-α and IL-6 was increased by 21.1-fold and 6.6-fold, respectively, in the FA-treated group (Figure 3A). In animals receiving AD-114, significantly lower TNF-α (68% reduction) and IL-6 (55% reduction) mRNA levels were observed (Figure 3A, *P* < 0.05). Consistent with this finding, ELISA of serum IL-6 levels indicated a significant decrease of serum IL-6 (36% reduction) in mice administered AD-114 compared with FA only (Figure 3B, *P* < 0.05). These data suggested that i-body AD-114 prevented the production of inflammatory TNF-α and IL-6 in FA-induced kidney fibrosis in mice.

Significant macrophage infiltration is a prominent feature in biopsy specimens of patients with CKD and indisputably plays a key role in kidney fibrosis (30–32). Macrophages can switch between classical M1 and alternative M2 phenotypes depending on the local microenvironment. Excessive or aberrant M1 or M2 macrophage activity can cause fibrosis. Interestingly, M2 macrophages express 16-fold greater CXCR4 mRNA levels in human peripheral blood-derived macrophages (33). Thus, activated macrophages contribute to the upregulation of CXCR4. This upregulation of CXCR4 on macrophages enables the SDF-1/CXCR4 interaction and thus the migration of macrophages to the diseased kidney. On the other hand, the upregulation of CXCR4 makes activated macrophages important targets of i-body AD-114. To determine if AD-114 regulates macrophage infiltration and activation, 1 macrophage marker — F4/80 — and 2 classical M1 activation markers — inducible NOS (iNOS) and interleukin-1β (IL-1β) — as well as 2 markers of alternative M2 activation that are associated with fibrosis — macrophage scavenger receptor (Msr1) and mannose receptor C type 1 (Mrc1) — were analyzed in kidney tissues. RT-PCR data demonstrated that the gene expression of F4/80, iNOS, and Mrc1 was increased by 8.9-fold, 8.7-fold, and 15.4-fold in the FA group and reduced by AD-114 by 54%, 69%, and 58%, respectively (Figure 3C, *P* < 0.05). AD-114 did not exhibit statistically significant inhibition of IL-1β and Msr1 mRNA levels, although the levels were elevated by 4.1-fold and 17.7-fold after FA treatment and trended toward decreases when treated with AD-114 (Figure 3D). These data suggest that the i-body AD-114 inhibits macrophage infiltration and attenuates some but not all markers of their activation.

### I-body AD-114 mitigates the upregulation of TGF-β1 and lysyl oxidase-like 2 in FA-induced kidney fibrosis.

**Figure 4:**
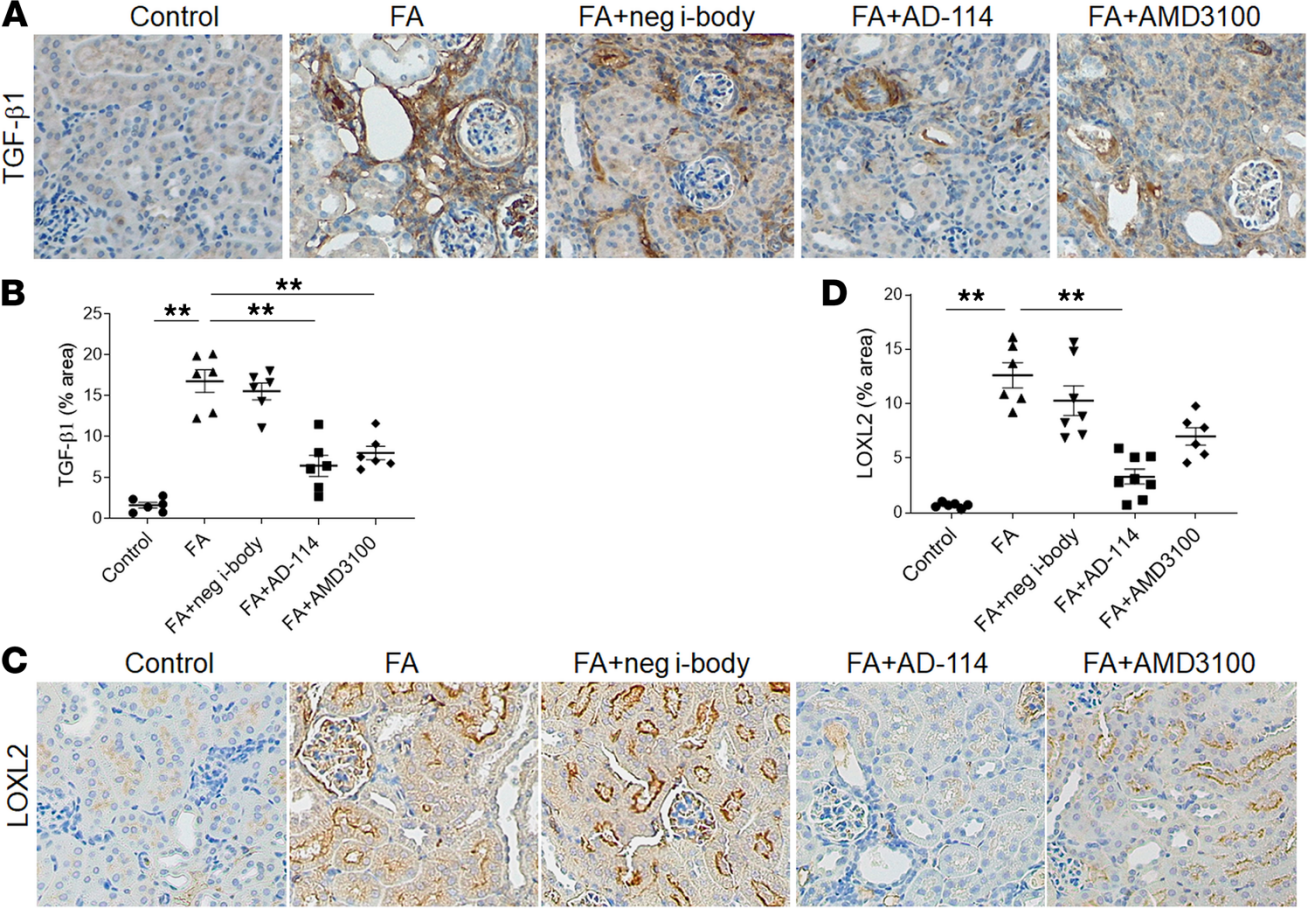
I-body AD-114 mitigates the upregulation of TGF-β1 and lysyl oxidase-like 2 in FA-induced kidney fibrosis. Representative images of (**A**) TGF-β1–stained and (**C**) lysyl oxidase-like 2–stained (LOXL2-stained) kidney section. Quantitation of (**B**) TGF-β1 and (**D**) LOXL2 immunohistochemical staining. Original magnification: ×200 in all. Statistical analysis was performed using 1-way ANOVA followed by Tukey’s multiple comparisons test. Results are presented as mean ± SEM. **P < 0.01. *n* = 6–8.

TGF-β1 has been considered a master regulator of renal fibrosis. Elevated TGF-β1 in the kidney is associated with the progression of FA-induced renal fibrosis (34). TGF-β1 induces renal fibrosis through induction of ECM deposition and myofibroblast activation as well as suppression of ECM degradation (35). Although a variety of cell types produce TGF-β1, several studies have identified macrophages as a critical source of TGF-β1 (36). Thus, next we evaluated if AD-114 mitigated TGF-β1 level in kidneys after exposure to FA (Figure 4). FA resulted in considerably increased TGF-β1–positive staining, whereas this effect was significantly reversed by 61% in the kidneys of AD-114–treated mice (Figure 4, A and B, *P* < 0.01).

ECM is a highly dynamic structure that is in constant flux of remodeling through synthesis and degradation of matrix. Dysregulation of this tightly regulated balance will result in excessive matrix deposition and fibrotic tissue formation (37). LOXL2, which is induced by TGF-β1, is a member of the lysyl oxidase family. It plays a critical role in ECM stabilization by facilitating collagen cross-links, myofibroblast activation, and epithelial-mesenchymal transition (EMT). Hence, the effect of AD-114 on the expression of LOXL2 was investigated. A dramatic increase in LOXL2 staining was observed in the kidneys of the FA group, whereas significantly less staining (73% reduction) was detected in kidneys from AD-114–treated mice (Figure 4, C and D, *P* < 0.01). Together, these data show that AD-114 mitigated the upregulation of profibrotic factor TGF-β1 and LOXL2.

### I-body AD-114 suppresses fibroblast activation in FA-induced kidney fibrosis.

**Figure 5:**
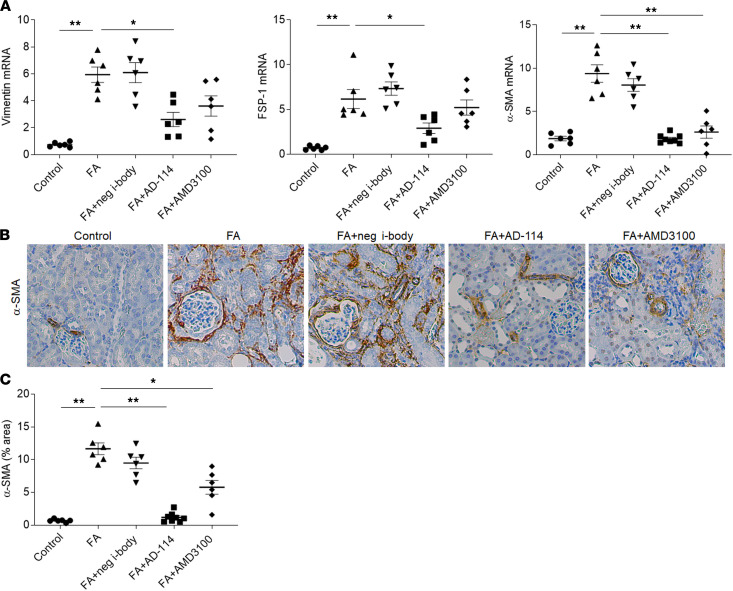
I-body AD-114 suppresses fibroblast activation in FA-induced kidney fibrosis. (**A**) mRNA expression of vimentin, FSP-1, and α-SMA in mouse kidneys was measured by quantitative RT-PCR. β-Actin was used as the endogenous control gene. (**B**) Representative images of α-SMA–stained kidney sections. (**C**) Quantitation of α-SMA immunohistochemical staining. Original magnification: ×200. Statistical analysis was performed using 1-way ANOVA followed by Tukey’s multiple comparisons test. Results are presented as mean ± SEM. *P < 0.05, **P < 0.01. *n* = 6–8.

Myofibroblast activation is a dominant feature of kidney fibrosis. A role for SDF-1 and CXCR4 in myofibroblast activation has been previously shown (23, 38, 39). Hence, we sought to clarify if AD-114 can reduce myofibroblast activation after exposure to FA. To characterize the role of AD-114 in the regulation of fibroblast activation, we examined markers of myofibroblasts, including vimentin, fibroblast-specific protein-1 (FSP-1), and α-smooth muscle actin (α-SMA) in kidney tissues. RT-PCR analyses of kidney tissues demonstrated that the expression of vimentin, FSP-1, and α-SMA was increased by 7.1-fold, 7.8-fold, and 4.1-fold, respectively, in FA-challenged mice (Figure 5A, *P* < 0.01). Treatment with AD-114 significantly decreased vimentin, FSP-1, and α-SMA expression by 55%, 53%, and 80%, respectively, compared with the FA group (Figure 5A, *P* < 0.05). Consistently, histopathologic analyses revealed augmented α-SMA staining after FA, and AD-114 administration reduced α-SMA protein level by 91% (Figure 5, B and C, *P* < 0.01).

### Delayed administration of i-body AD-114 also attenuates renal fibrosis.

We then tested whether delayed administration of AD-114 is also effective in reducing renal fibrosis, a scenario that is more relevant to the clinical setting. As shown in Supplemental Figure 4, negative control i-body 21H5, AD-114, or AMD3100 was administrated to mice starting at day 7 after FA injection, a time point when significant kidney injury was already established as demonstrated in Figure 1, G and H.

**Figure 6:**
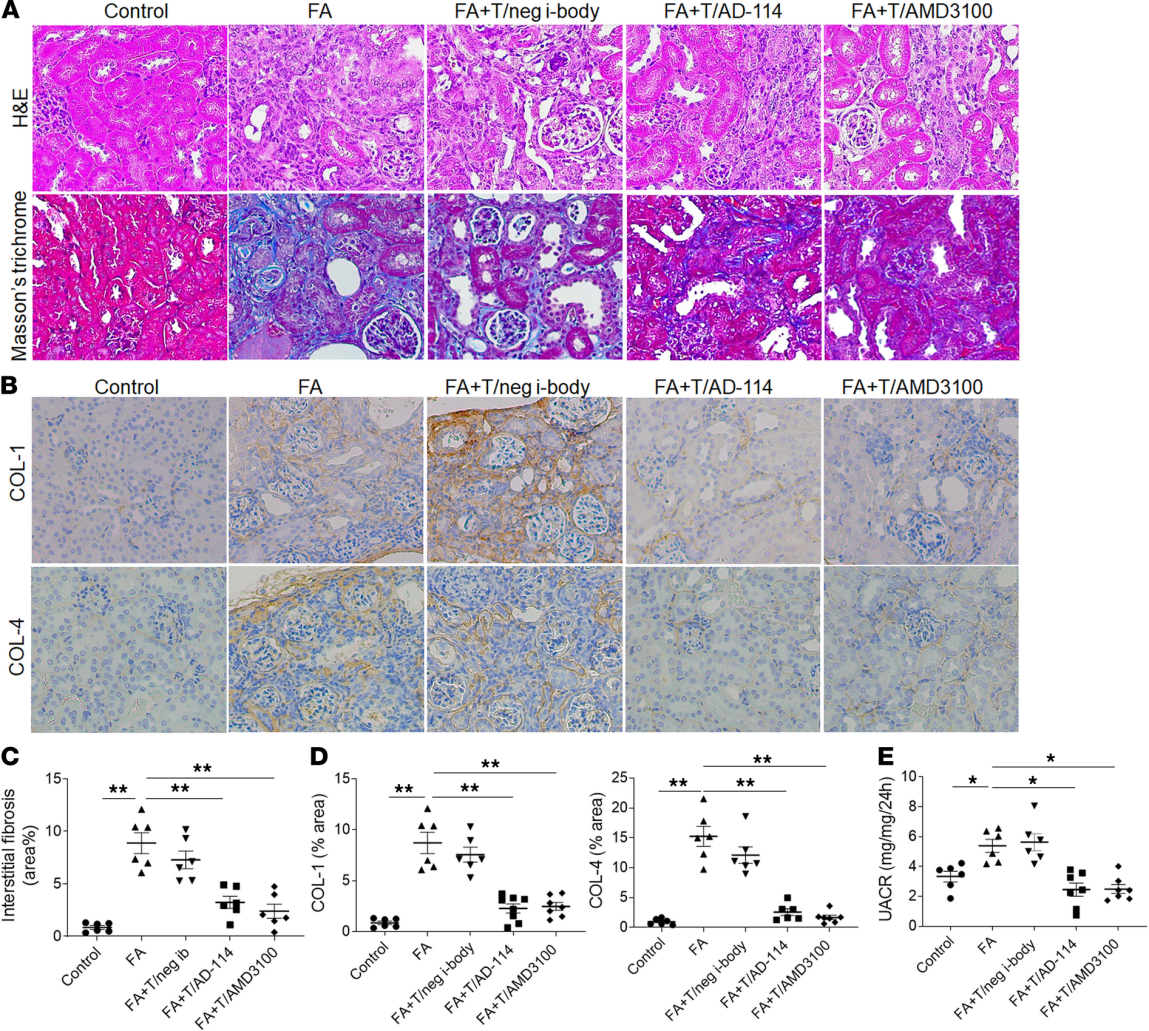
Late administration of AD-114 attenuates FA-induced renal fibrosis and improves kidney function in the therapeutic mouse model. Mice were dosed daily with negative i-body, AD-114, and AMD3100 from days 7–21. (**A**) Representative images of H&E and Masson’s trichrome staining. (**B**) Representative images of COL-1– and COL-4–stained kidney sections. (**C**) Quantitative analysis of tubulointerstitial fibrosis from Masson’s trichrome staining using ImageJ software. (**D**) Quantitation of COL-1 and COL-4 immunohistochemical staining. (**E**) A 24-hour urine was collected at day 21, and urinary albumin and creatinine levels were detected for UACR calculation. To differentiate the preventative study, the groups targeted for therapeutic intervention are prefaced by “T/.” Original magnification: ×200 in all. Statistical analysis was performed using 1-way ANOVA followed by Tukey’s multiple comparisons test. Results were presented as mean ± SEM. *P < 0.01, **P < 0.01. *n* = 6–8.

Mice that were administered FA and the various treatment interventions from days 7–21 were euthanized, and kidneys were analyzed by H&E and Masson’s trichrome staining. Figure 6A displays representative images from each group. As demonstrated by H&E and Masson’s trichrome staining, the kidney demonstrated typical features of renal fibrosis in the FA and FA+negative i-body groups. In contrast, daily AD-114 treatment from day 7 significantly decreased the severity of tubulointerstitial fibrosis by 64% (Figure 6C, *P* < 0.01).

Consistently, FA-treated mice showed substantially increased deposition of COL-1 and COL-4 in the interstitial area of kidney cortex, whereas this effect was significantly reversed by 74% and 83%, respectively, in mice with daily treatment of AD-114 from days 7 to 21 (Figure 6, B and D, *P* < 0.01). These data indicate that i-body AD-114 can suppress ECM overproduction and reduce renal fibrosis in an established mouse model of FA nephropathy. Concomitantly, kidney function measurement indicated that UACR was considerably elevated in FA mice, and this impairment was significantly attenuated by 47% after daily injection of AD-114 from days 7 to 21 (Figure 6E, *P* < 0.05).

### I-body AD-114 binds TGF-β1–induced CXCR4 on RPTEC/TERT1 cells and inhibits TGF-β1–induced ECM overexpression.

Renal tubular cells are the most abundant cells in kidneys. They play fundamental roles during kidney injury. Although CXCR4 is expressed in various types of cells, it is mainly observed in tubular cells, especially PTCs (13, 40). To further evaluate the effect of i-body AD-114 on human PTCs, the RPTEC/TERT1 cell line was used. It has been confirmed that the RPTEC/TERT1 cell line exhibits functional similarity with in vivo PTCs (41, 42).

**Figure 7:**
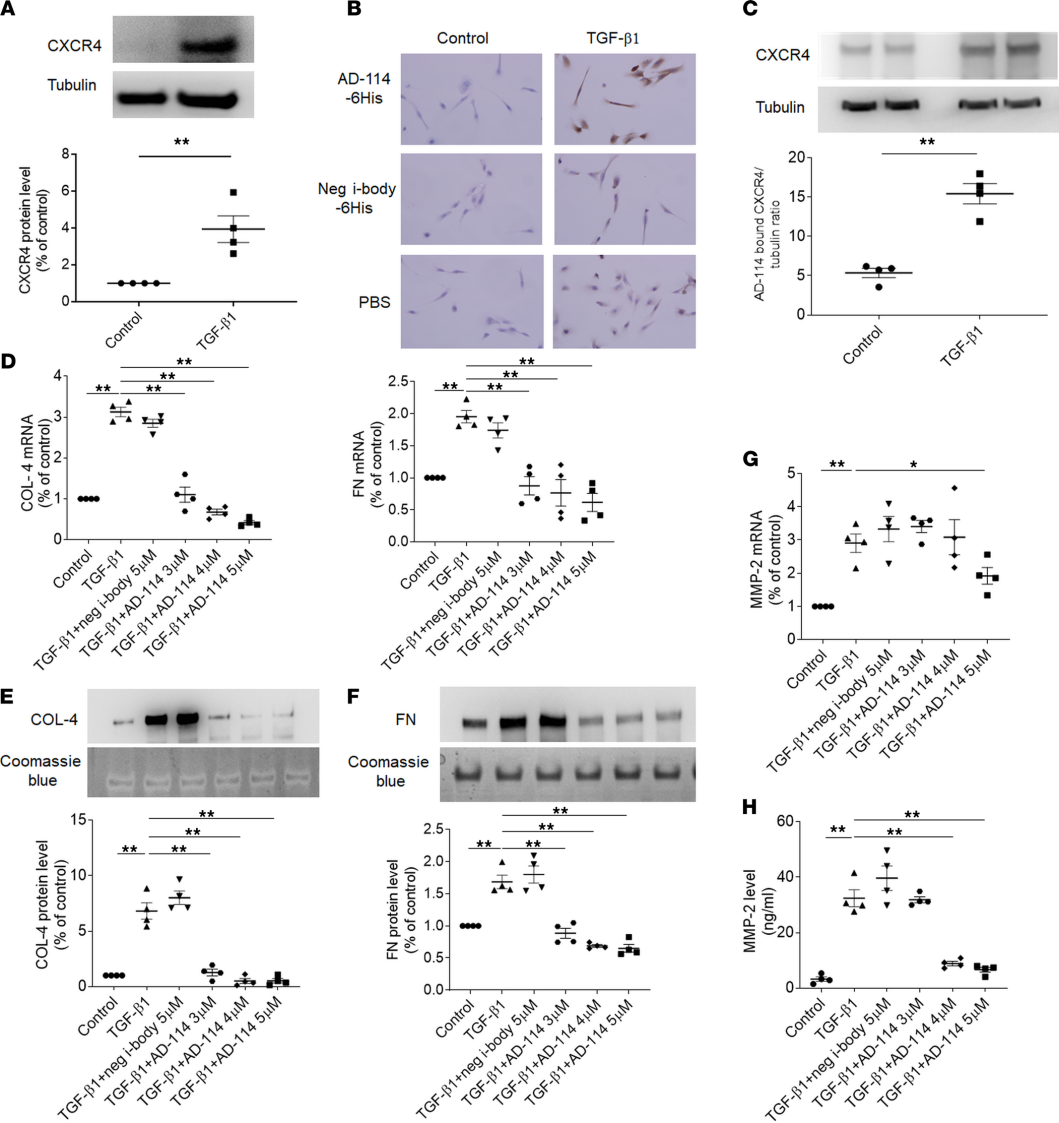
I-body AD-114 binds TGF-β1–induced CXCR4 on RPTEC/TERT1 cells and inhibits TGF-β1–induced ECM overexpression. (**A**–**C**) RPTEC/TERT1 cells were incubated with/without TGF-β1 (2 ng/mL) for 48 hours. (**A** and **C**) Cell lysates were analyzed by Western blot. Data are mean ± SEM and analyzed by Student’s unpaired 2-tailed *t* test. *n* = 4. (**B**) Cells were grown on glass slides and analyzed by ICC. In **B** and **C**, AD-114-6His and negative control i-body 21H5-6His were used as primary antibodies. Original magnification: ×400. (**D**–**H**) Cells were exposed to TGF-β1 with/without AD-114 for 48 hours. (**D**) Gene expression of COL-4 and FN was analyzed by quantitative RT-PCR. (**E** and **F**) Supernatants were collected and COL-4 and FN were analyzed by Western blot. MMP-2 expression was analyzed by (**G**) quantitative RT-PCR and (**H**) ELISA. Statistical analysis was performed using 1-way ANOVA followed by Tukey’s multiple comparisons test. The data are presented as mean ± SEM. *P < 0.05, **P < 0.01, *n* = 4.

TGF-β1 is known to increase protein levels and surface expression of CXCR4 in certain human cells (43, 44). RPTEC/TERT1 cells were incubated with or without TGF-β1 (2 ng/mL) for 48 hours, and cell lysates were collected for Western blot analysis. As expected, CXCR4 levels in RPTEC/TERT1 were significantly lower in the absence of TGF-β1, while exposure of RPTEC/TERT1 cells to TGF-β1 resulted in significantly increased CXCR4 expression by 2.9-fold (Figure 7A, *P* < 0.01).

To further determine if the i-body AD-114 can bind the upregulated CXCR4 in RPTEC/TERT1 cells, immunocytochemistry (ICC) and Western blot were performed with AD-114-6His (AD-114 tagged with 6 consecutive histidine residues) or negative control i-body 21H5-6His (21H5 tagged with 6 consecutive histidine residues) as primary antibodies. In Figure 7B, AD-114-6His specifically bound TGF-β1–induced CXCR4 on RPTEC/TERT1 compared with 21H5-6His. Consistently, TGF-β1 significantly increased CXCR4 protein expression by 1.8-fold, which was specifically bound by AD-114 (Figure 7C, *P* < 0.01). These data clearly indicate that the i-body AD-114 can bind RPTEC/TERT1 cells specifically through TGF-β1–induced CXCR4.

As AD-114 can bind RPTEC/TERT1 cells, we further evaluated the impact of AD-114 on TGF-β1–induced ECM expression in RPTEC/TERT1. Our study has shown that for RPTEC/TERT1 cells, AD-114 in a concentration of 2 μM or lower does not inhibit the TGF-β1–induced COL-4 and FN upregulation. Hence the i-body AD-114 was added to cell culture at 3 μM, 4 μM, and 5 μM in all experiments described below.

As shown in Figure 7D, TGF-β1 induced upregulation of COL-4 and FN genes compared with control. Coincubation of AD-114 at 3 μM significantly inhibited TGF-β1–induced COL-4 mRNA by 66% and FN mRNA by 55% compared with the negative control i-body (Figure 7D, *P* < 0.01). To further investigate the effect of AD-114 on ECM protein secretion of RPTEC/TERT1 cells, Western blot was performed to detect the protein levels of COL-4 and FN. TGF-β1 induced significant increases in COL-4 and FN secretion (Figure 7, E and F, *P* < 0.01). Incubation with AD-114 3 μM reduced the secretion of COL-4 and FN by 81% and 47%, and the inhibitory effect increased with the elevation of the i-body concentration (Figure 7, E and F, *P* < 0.01). These data show that TGF-β1 induced fibrotic responses in RPTEC/TERT1 cells and that such responses can be reversed by concomitant inhibition of CXCR4 by the i-body AD-114.

MMP families are zinc-dependent enzymes capable of cleaving components of the ECM and thus crucial in the development of kidney fibrosis (45). MMP-2 is a major member of the MMP families, regulating ECM turnover following TGF-β1 exposure (46–48). Hence, we sought to determine in RPTEC/TERT1 cells the effects of the i-body AD-114 on the gene expression of MMP-2. As shown in Figure 7G, TGF-β1 increased MMP-2 mRNA levels by 3.5-fold, and coincubation of RPTEC/TERT1 cells with AD-114 (5 μM) inhibited the induction of MMP-2 mRNA (34% reduction, *P* < 0.05) by TGF-β1. The secretion of MMP-2 was further assessed by ELISA. As shown in Figure 7H, the significant induction of MMP-2 secretion by TGF-β1 (45-fold increase) was inhibited by 4 μM AD-114 (73% reduction, *P* < 0.01).

### I-body AD-114 blocks CXCR4 signaling via p38 MAPK and PI3K/AKT/mTOR signaling pathway in RPTEC/TERT1 cells.

**Figure 8:**
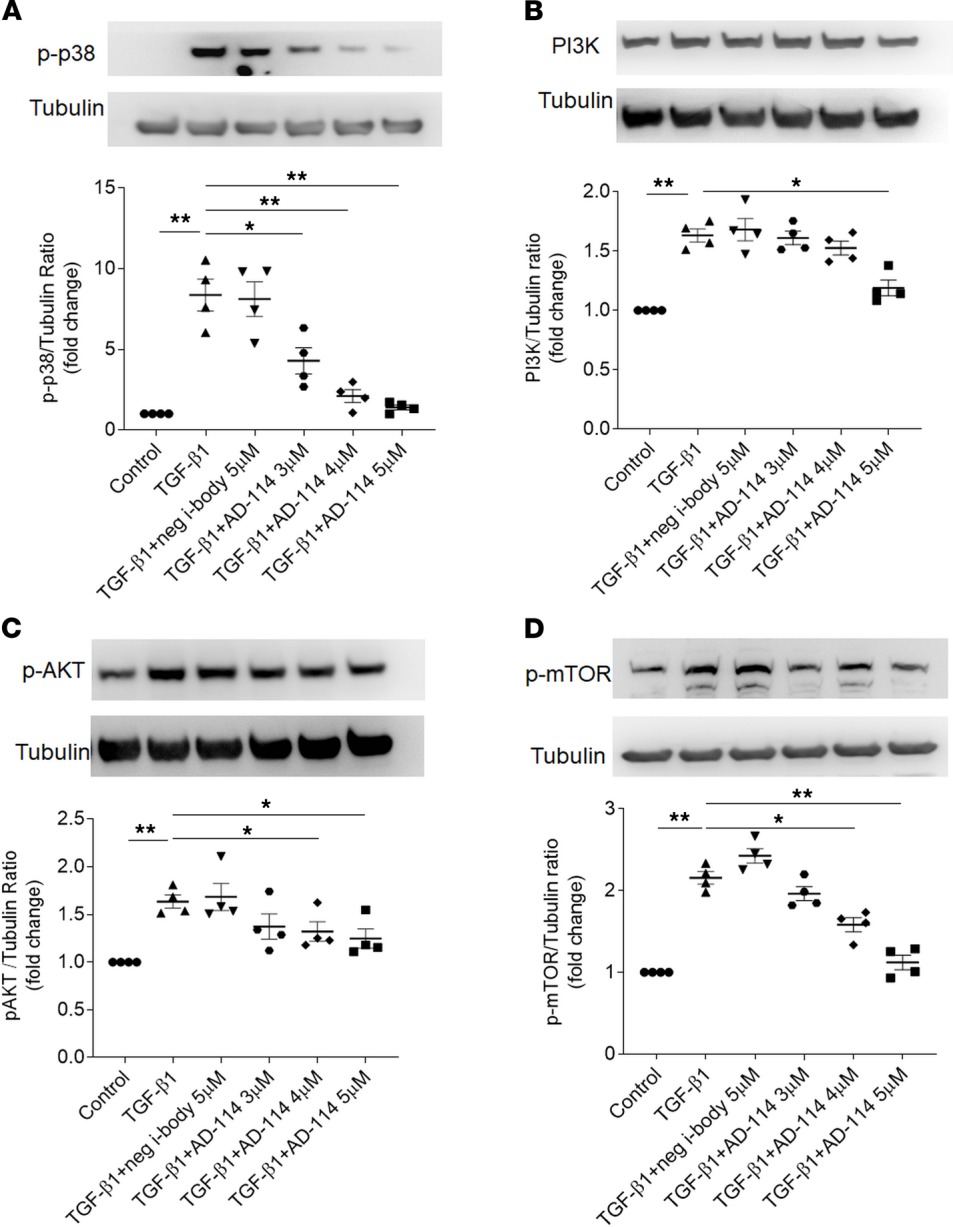
I-body AD-114 blocks CXCR4 signaling via p38 MAPK and PI3K/AKT/mTOR signaling pathway. RPTEC/TERT1 cells were treated with TGF-β1 with or without i-body AD-114 for 48 hours. Lysis proteins were collected and expression of (**B**) PI3K and phosphorylation of (**A**) p38 MAPK, (**C**) AKT, and (**D**) mTOR was analyzed by Western blot. Results are presented as mean ± SEM. Statistical analysis was performed using 1-way ANOVA followed by Tukey’s multiple comparisons test. * *P* < 0.05, **P < 0.01, *n* = 4.

It is reported that CXCR4 stimulation activates p38 MAPK and PI3K/AKT/mTOR signaling in tubular epithelial cells (49). To further understand the mechanism whereby AD-114 influences CXCR4 signaling in RPTEC/TERT1 cells, the effects of AD-114 on CXCR4 signaling transduction pathways were investigated. As shown in Figure 8, exposure of RPTEC/TERT1 cells to TGF-β1 resulted in significantly increased p38 MAPK (Figure 8A, *P* < 0.01), AKT (Figure 8C, *P* < 0.01), and mTOR (Figure 8D, *P* < 0.01) phosphorylation. Concurrent exposure to AD-114 inhibited the TGF-β1–mediated increases in phosphorylation of p38 MAPK, AKT, and mTOR by 70%, 20%, and 25%, respectively, at 4 μM (Figure 8, A, C, and D, *P* < 0.05), with the inhibition becoming more marked with increasing concentration of AD-114. PI3K expression was not inhibited until the concentration of AD-114 reached 5 μM (24% reduction, Figure 8B, *P* < 0.05), indicating that AD-114 may have a dominant effect on downstream signaling of PI3K.

### Administration of Fc-fused AD-114, AD-214, ameliorates UUO-induced kidney fibrosis.

The fusion of an Fc fragment at the C-terminus of AD-114 resulted in dimer formation, which was reflected in an enhanced affinity to CXCR4 of low picomolar (Supplemental Figure 5A). AD-214 has been shown to bind to CXCR4 overexpressed in CHO cells as well as endogenous CXCR4 on T cells (Supplemental Figure 5, B–D).

CXCR4 is markedly upregulated in kidneys that have undergone ureteric obstruction. To analyze whether the renoprotective effect of i-body AD-114 is generalizable to other forms of renal fibrosis, we used an additional model, the UUO model. The day following the UUO procedure, mice were dosed i.p. with negative control i-body 21H5-Fc and AD-214 and were then dosed once every second day until day 14. As demonstrated by H&E staining in Supplemental Figure 5E, UUO kidneys displayed severe morphologic lesions characterized by tubular dilation, atrophy, inflammatory cell accumulation, and tubulointerstitial fibrosis. Conversely, the kidneys of animals treated with AD-214 exhibited considerably fewer morphologic abnormalities. Consistently, IHC staining revealed that UUO-induced deposition of fibrotic marker COL-1 was significantly attenuated (Supplemental Figure 5, E and F, *P* < 0.05) with AD-214 treatment. These data show that AD-214 is renoprotective in UUO-induced renal fibrosis.

## Discussion

I-bodies are a class of humanized next-generation antibodies with the potential to overcome some of the limitations of monoclonal antibodies in therapeutics (22). This study was undertaken to evaluate the antifibrotic effect of anti-CXCR4 i-body AD-114 in kidney fibrosis and to elucidate the possible underlying mechanisms by utilizing the in vivo toxin-induced FA nephropathy model of CKD and the in vitro human PTC line.

The i-body AD-114, which specifically antagonizes CXCR4, was expressed in *E*. *coli* or *Pichia* *pastoris* in 3 C-terminal formats: AD-114-6H, AD-114-Im-FH, and AD-114-PA600-6H (23, 50). In our study, AD-114 was applied in the form of AD-114-PA600-6H as it has the longest half-life (7.77 hours). It remains in the bloodstream of mice for 72 hours (23). In a mouse model of pulmonary fibrosis, daily prophylactic treatment with AD-114 i.p. for 21 days markedly ameliorated fibrotic lung remodeling relative to control groups (23). In the FA nephropathy model, FA leads to acute injury followed by fibrosis with elevated fibrotic markers (TGF-β1 and α-SMA) after 6 days and tubulointerstitial fibrosis after 14 days. As reported, high dosage of FA induced tubular necrosis in the acute phase (from 7 days) (51) and robust interstitial fibrosis in the chronic phase (from 21 days) (52). Thus, to assess the preventative effect of AD-114 on kidney fibrosis in this study, mice were administered AD-114 from the day of FA injection and administration continued for 21 days.

In this study, we demonstrated that CXCR4 was upregulated in fibrotic kidneys and administration of anti-CXCR4 i-body AD-114 prevented and furthermore reversed kidney fibrosis in the mouse model of FA nephropathy, as evidenced by reduced fibrosis, reduced ECM protein expression, and attenuation of FA-mediated increased UACR. Previous reports have shown that chronically high CXCR4 expression in multiple effector cell types can contribute to the pathogenesis of renal fibrosis by altering their biological profile, and crosstalk between CXCR4 and TGF-β1 pathways contributes to the progression of renal fibrosis (13). Consistent with this report, we observed AD-114 administration correlated with multiple potentially beneficial mechanisms that limit or reverse renal fibrosis.

Chronic inflammation plays a unique role in the pathophysiology of CKD. Chronic inflammation in the kidney creates a milieu for inducing sustained CXCR4 expression in multiple cell types, including neutrophils, monocytes, macrophages, and tubular cells (13). CXCR4 participates in proinflammatory cytokine production and inflammatory cell infiltration (53, 54). Inflammatory cells infiltrating the renal interstitium play a major role in the initiation and progression of tubulointerstitial fibrosis (55). The degree to which a number of cell types, including macrophages, DCs, T and B cells, and NK cells, accumulate in the renal interstitium correlates with the extent of fibrosis. Macrophages are found in close proximity to collagen-producing myofibroblasts and predominate in the cellular infiltrate in fibrosis. Previous studies in a murine air-pouch model showed an inhibitory effect of AD-114 on leukocyte migration, indicating that AD-114 is able to block CXCR4-expressing immune cells’ infiltration in the kidney. In this study, we demonstrated that mice administered AD-114 had a significant reduction in macrophage infiltration as well as activation as evidenced by decreased F4/80, iNOS, and Mrc1 (*P* < 0.05). The reduced numbers and activity of macrophages are likely due to the indirect effect of blocking leukocyte recruitment and/or direct inhibition on macrophage infiltration through antagonizing SDF-1/CXCR4 interaction, which requires further investigation.

TGF-β1 is the primary stimulator that drives fibrosis through activation of myofibroblasts, induction of ECM, and inhibition of ECM degradation (35). The macrophage is a major cell type producing TGF-β1. Following translation, ECM molecules, such as collagens, undergo several posttranslational modifications that facilitate their cross-linking and consequently the formation of fibers (56). A key enzyme in this process is LOXL2, an enzyme that can be induced by TGF-β1. LOXL2 affects fibrosis irreversibly through promoting cross-linkage of collagen fibers in the ECM (57). In the present study, expression levels of TGF-β1 and LOXL2 were upregulated in fibrotic kidneys of FA-treated mice, and these changes were reversed by administration of AD-114. Thus, we suggest that the inhibition of FA-induced renal fibrosis by AD-114 is at least partially mediated by antagonizing TGF-β1 and LOXL2. The reduced TGF-β1 and LOXL2 could result from reduced leukocyte recruitment and/or decreased macrophage activity, but this mechanism remains elusive and warrants future study.

As the effector cells of TGF-β1, activated myofibroblasts are the cells most responsible for interstitial expansion and matrix accumulation during renal fibrosis (58). Recently, the significant role of CXCR4 signaling in myofibroblast activation has been recognized (13, 38, 59, 60). CXCR4 signaling is thought to mediate the recruitment of CXCR4^+^ fibrocytes to the injured organ (59, 60) or promote myofibroblast differentiation (38). In this study, we found FA increased the number of α-SMA–positive renal fibroblasts, while AD-114 significantly suppressed the activation of kidney fibroblasts, paralleled by a substantial reduction in ECM. Vimentin and FSP-1 are another 2 markers for fibroblast activation. Enhanced mRNA expression of vimentin and FSP-1 by FA was also inhibited by AD-114. Vimentin is also a typical mesenchymal marker, suggesting that AD-114 can at least partially block epithelial dedifferentiation. Collectively, these data implied that injury-activated myofibroblasts are important targets of i-body AD-114. We propose that rather than simply blocking the interaction between CXCR4 and its ligand SDF-1, AD-114 can bind to CXCR4 and modulate intracellular downstream signaling that are involved in fibroblast activation.

Besides immune cells, CXCR4 is mainly expressed in tubular cells. Intense CXCR4 staining can be observed in tubular segments of human biopsies of CKD (8). In this study, we demonstrated that AD-114 can suppress TGF-β1–induced overexpression of ECM in RPTEC/TERT1 cells through inhibiting CXCR4 downstream signaling pathways, PI3K/AKT/mTOR and p38 MAPK, which also constitute the noncanonical signaling pathway of TGF-β1. Previous studies have shown that CXCR4 is required for TGF-β–induced cell migration while inhibition of the TGF-β receptor 1 attenuates CXCR4 expression (61). The p38 MAPK pathway is an important intracellular signaling pathway involved in the production of profibrotic mediators (62). It is reported that inhibition of p38 MAPK reduces renal fibrosis in various models of renal fibrosis (63–67). We have shown that dysregulation of the PI3K/AKT/mTOR signaling pathway mediates impaired autophagy in a diabetic model of renal fibrosis (68). Additionally, ROS induces EMT via the TGF-β1/PI3K/AKT/mTOR pathway in diabetic nephropathy (69), and tamoxifen ameliorates obstructive nephropathy through Src and the PI3K/AKT/mTOR pathway (70). Thus, we predict that a crosstalk or a regulatory circuit may exist between the TGF-β and CXCR4 pathways, which is limited by AD-114. However, as this is investigated in an in vitro model, it cannot exactly mimic the in vivo events happening during CKD as there are no other cells included.

**Figure 9:**
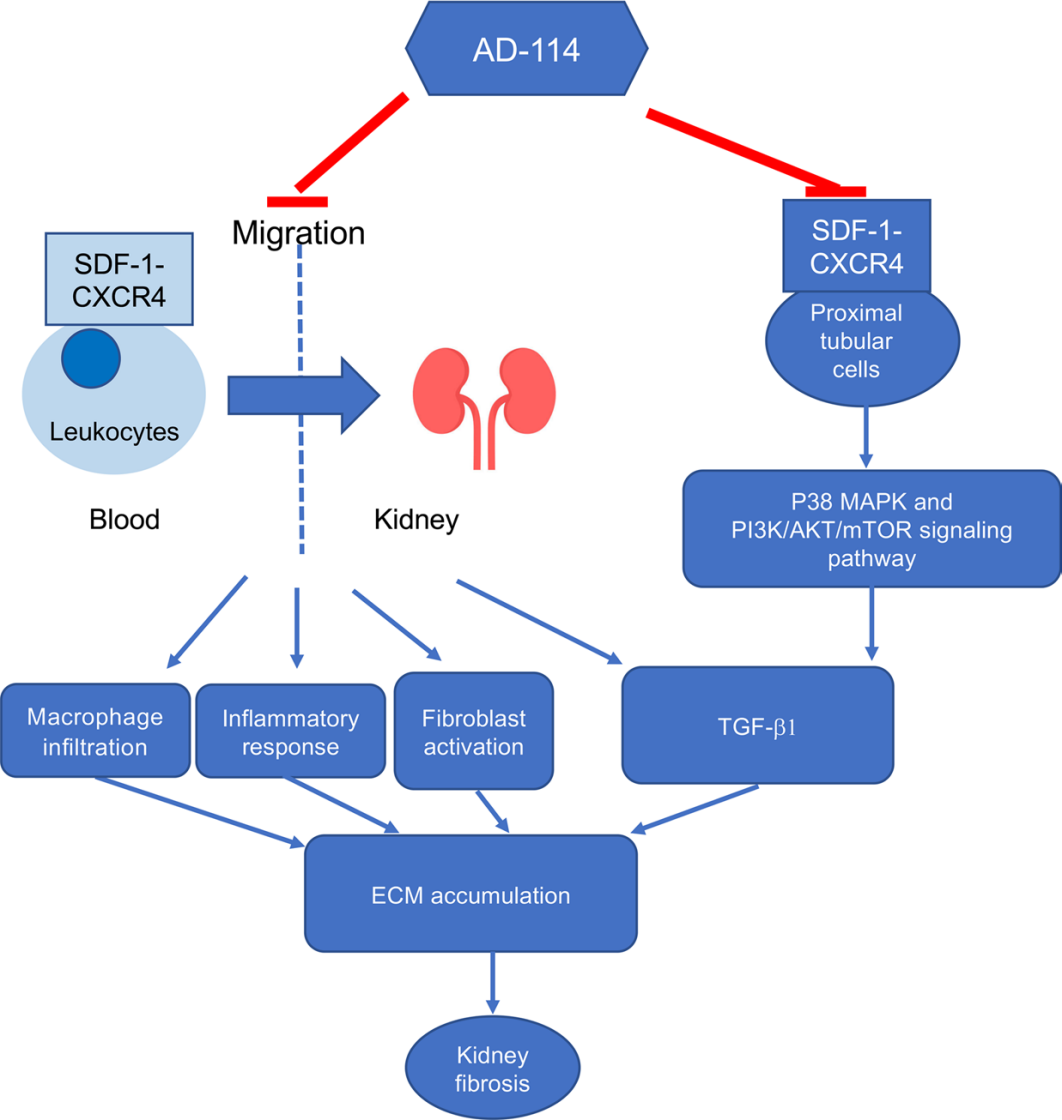
Proposed mechanisms of how AD-114 ameliorates kidney fibrosis. AD-114 blocked leukocyte migration, leading to reduced inflammatory cytokines and macrophage infiltration, and mitigated TGF-β1 upregulation as well as fibroblast activation. AD-114 also blocked CXCR4 downstream p38 MAPK and PI3K/AKT/mTOR signaling in PTCs. All of these resulted in the inhibition of ECM accumulation and thus attenuated kidney fibrosis.

These studies provide a mechanistic understanding of the role of AD-114 in the inhibition and reversal of kidney fibrosis. CXCR4 is expressed in both immune cells and tubular cells. This i-body targeting CXCR4 reduces inflammatory cytokine and macrophage infiltration and mitigates TGF-β1 upregulation, as well as suppresses fibroblast activation. The results are consistent with previous findings that AD-114 reduces immune cell migration to the diseased organ as it inhibits the innate chemotactic function of CXCR4 through binding to critical residues in CXCR4. Our results further indicate that AD-114 blocked p38 MAPK and PI3K/AKT/mTOR downstream of CXCR4 signaling in PTCs, which contributed to the inhibition of fibrotic markers (Figure 9). These results suggest that AD-114 (and potentially AD-214) not only antagonizes the interaction between CXCR4 and its ligand SDF-1 but also regulates CXCR4 intracellular downstream signaling.

We then examined the therapeutic effect of AD-114 at an early time point in the development of renal fibrosis in a mouse model of FA nephropathy, where AD-114 administration commenced 7 days after FA injection. At this time point, mice exhibited typical fibrotic changes in Masson’s trichrome staining as well as increases in ECM deposition and renal structural injury. Daily AD-114 administration to FA-treated mice from this time point resulted in an attenuated degree of fibrosis as determined 21 days later at the endpoint of this study.

Although the antifibrotic potential of i-body AD-114 (and AD-214) is convincing, there are several limitations. First, since there are many effector cells and cytokines involved in kidney fibrosis, further investigation is required to better understand the mechanisms of CXCR4 signaling. Second, as the therapeutic study commenced from day 7, which is a relatively early intervention in the development of kidney fibrosis, a more delayed intervention using AD-114 is warranted.

To conclude, this study is the first to our knowledge to report that i-bodies targeting CXCR4 ameliorate kidney fibrosis in both FA and UUO models of kidney disease. These data suggest that AD-114 has potential utility for therapeutic use in reversing kidney fibrosis. Because existing therapies are at best minimally ineffective and other CXCR4 antagonists, such as AMD3100, have been demonstrated to exert significant toxicity with off-target effects, these findings are of obvious clinical significance.

## Methods

### Kidney biopsy collection.

To determine CXCR4 expression in fibrotic kidneys, kidney biopsies were collected from normal nephrectomy specimens and from patients with DKD and kidney fibrosis recruited from Royal North Shore Hospital, Sydney, New South Wales, Australia. Kidney samples from STZ-induced eNOS^–/–^ DKD mice, FA mice, and UUO mice were derived within our research group.

### I-body and CXCR4 antagonist AMD3100.

I-body AD-114-PA600 (AD-114) and AD-114-PA600-6His (AD-114-6His, AD-114 tagged with 6 consecutive histidine residues) were produced using PASylation technology in collaboration with XL-protein GmbH (50). AD-214 (AD-114 was fused at its C-terminus with a human IgG1 mutant Fc region) and nonspecific negative control i-bodies 21H5-Im7-FH (21H5), 21H5-Im7-FH-6His (21H5-6His, 21H5 tagged with 6 consecutive histidine residues), and 21H5-Fc (21H5 was fused at its C-terminus with a human IgG1 mutant Fc region) were supplied by AdAlta Limited. AMD3100, which is an inhibitor of CXCR4 approved for applications in stem cell mobilization (71), was purchased from Tocris Bioscience, Bio-Techne, as the octahydrochloride salt and used as a positive control.

### Animal studies.

Male C57BL/6 mice, 6–8 weeks old, weighing 20–25 g, were randomized by body weight and divided into treatment groups (*n* = 6–8 per group). Mice were purchased from the Kearns Facility, Kolling Institute, Sydney, New South Wales, Australia.

For animal study 1, FA nephropathy was experimentally induced at day 0 by i.p. injection of a single dose of FA (250 mg/kg in the vehicle of 0.3 M NaHCO_3_). To evaluate CXCR4 expression at different time points after FA injection, mice were humanely euthanized at days 3, 5, 7, 14, and 21.

For animal study 2 (preventative and therapeutic studies), to evaluate the effects of i-body AD-114, FA nephropathy was induced at day 0 as above. For the preventative study, mice were dosed i.p. 1 hour prior to FA injection with negative i-body (10 mg/kg), AD-114 (10 mg/kg), or AMD3100 (10 mg/kg) and were then dosed daily with these treatments until day 21 (Supplemental Figure 2). To assess the therapeutic potential of the i-body AD-114 on established kidney injury (therapeutic study), a second batch of FA-treated mice were dosed daily with negative i-body (10 mg/kg), AD-114 (10 mg/kg), and AMD3100 (10 mg/kg) from days7–21 (Supplemental Figure 3). A preterminal 24-hour urine was collected in metabolic cages 1 day before the mice were humanely euthanized. Left kidneys were removed and snap-frozen in liquid nitrogen and subsequently stored at –80°C for the isolation of RNA, and right kidneys were fixed in 10% buffered formalin for histologic examination.

For animal study 3 (second model), male C57BL/6J mice, which were purchased from the Kearns Facility, underwent UUO according to published methods. The left ureter was isolated and tied off 0.5 cm from the pelvis while the right ureter was left unclamped and served as the sham-operated control. From the following day of UUO, negative i-body 21H5-Fc (5 mg/kg) and AD-214 (5 mg/kg) or AMD3100 (5 mg/kg) were administered i.p. once every 2 days until day 14. Kidneys were taken and fixed by immersion in 10% phosphate-buffered formalin for further analyses.

### Urinary albumin/creatinine measurement.

Urinary albumin was measured using a Mouse Albumin ELISA kit (Crystal Chem), and urinary creatinine was measured using a creatinine (urinary) colorimetric assay kit (Cayman Chemical), according to instructions provided by the manufacturer. Albuminuria was expressed as UACR.

### Serum IL-6 measurement.

Blood was collected and clotted for 30 minutes at room temperature before centrifugation for 15 minutes at 1000*g* at 4°C. The supernatant (serum) was then transferred into a clean tube. Serum IL-6 concentrations were measured by ELISA, according to the manufacturer’s instructions (Eagle Biosciences).

### Kidney histology and IHC.

Paraffin-embedded sections were used for histologic and IHC staining. Changes in renal morphology were examined by H&E staining. Matrix deposition within the interstitium was assessed using Masson’s trichrome staining. For IHC staining, kidney slides were incubated in citrate buffer (pH 6, heated to 99°C) for epitope retrieval and 0.3% hydrogen peroxide to block endogenous peroxidase activity. After preincubation with 10% protein block (Dako) to block nonspecific binding of antibodies, the tissues were incubated overnight at 4°C with primary antibodies against COL-1 (Abcam, 34710), α-SMA (MilliporeSigma, A2547), FN (Abcam, 45688), COL-4 (Abcam, 6586), COL-3 (Abcam, 7778), CXCR4 (Santa Cruz Biotechnology, sc-53534), TGF-β (Santa Cruz Biotechnology, sc-130348), and LOXL2 (Santa Cruz Biotechnology, sc-66950). Slides were then washed and incubated with secondary antibodies EnVision+System-HRP Labelled Polymer Anti-rabbit (Dako, K4003) and EnVision+System-HRP Labelled Polymer Anti-mouse (Dako, K4001). After washing with TBS-Tween 20, kidney sections were covered with DAB (Dako) for 10 minutes to produce a brown color. Ten randomly chosen fields of kidney cortex were captured per mouse, and staining was quantified as percentage of total area, using ImageJ.

### Cell culture.

RPTEC/TERT1 cells (ATCC CRL4031), a human PTC line that ectopically expresses the catalytic subunit of telomerase (TERT), were cultured according to the instructions from ATCC. Cells were seeded into 6-well plates and incubated with or without recombinant human TGF-β1 (2 ng/mL) in the presence or absence of AD-114 for 48 hours. Then the culture supernatants, total RNA, and cell lysates were collected.

### ICC.

RPTEC/TERT1 cells were seeded into 6-well plates with a coverslip in each well and grown with or without TGF-β1 (2 ng/mL) for 48 hours. The cells were fixed in 100% methanol (chilled at –20°C) at room temperature (RT) for 5 minutes. After washing, cells were blocked with 1% BSA and 22.52 mg/mL glycine in PBS with Tween 20 at RT for 1 hour. Then cells were incubated with the primary antibody AD-114-6His at 4°C overnight. After that, cells were washed and incubated with the secondary anti-6X His tag antibody (HRP) (Abcam, ab197049) for 1 hour at RT, followed by incubation with DAB, and counterstained with hematoxylin.

### RNA isolation and RT-PCR analysis.

Total RNA was extracted from cells or mouse kidney cortex tissues using the RNeasy Mini Kit (QIAGEN). The cDNA was synthesized with iScript cDNA synthesis kit (Bio-Rad). Quantitative RT-PCR was performed using the SYBR Green Master Mix (Bio-Rad) with the intron-spanning primers as shown in Supplemental Table 1 on the ABI Prism 7900 Sequence Detection System (Applied Biosystems). The relative mRNA expression level was calculated by ExpressionSuite software (Thermo Fisher Scientific). GAPDH and β-actin were used as endogenous control genes for human RPTEC/TERT1 cells and mice, respectively.

### Western blot analysis.

FN, COL-3, and COL-4 were measured in cell culture supernatant, and cell lysates were prepared in RIPA buffer with protease inhibitors (Roche). Samples were separated by SDS-PAGE, then transferred to Hybond ECL nitrocellulose membrane (Amersham). The membranes were incubated with primary antibodies at 4°C overnight followed by 1 hour of incubation with HRP-conjugated secondary antibody at room temperature. Anti-PI3K was purchased from BD Biosciences (catalog 610045). Anti–phospho-p38 MAPK (catalog 9211), anti-phospho-AKT (catalog 4060) were purchased from Cell Signaling Technology. Anti–phospho-mTOR (catalog ab109268) and anti–α-tubulin were from Abcam (catalog ab56676). The blots were then visualized with standard ECL methodology. α-Tubulin protein was used as the endogenous control.

### MMP-2 detection by ELISA.

The concentration of MMP-2 in cell culture supernatant was quantified using ELISA kits (MilliporeSigma) according to the manufacturer’s instruction. The concentration of samples was subsequently calculated using the standard curve and mean absorbance values for each sample.

### Kinetic binding assay.

Kinetic binding analysis of all AD-214 was confirmed by surface plasmon resonance as previously described by Griffiths et al. (22). CHO cells treated with 0.1 μg/mL tetracycline to induce CXCR4 expression or human CD8^+^ T cells were incubated for 10 minutes at room temperature and subsequently washed with PBS. The cells were then stained with F(ab′)_2_-FITC (Jackson ImmunoResearch Laboratories), washed, and fixed in 1% paraformaldehyde. The CytoFLEX S flow cytometer (Beckman Coulter) was utilized for flow cytometric analysis, and data were analyzed using FlowJo software (Tree Star Inc.).

### Statistics.

Data are shown as mean ± SEM. Statistical analysis of data from 2 groups was compared by Student’s unpaired 2-tailed *t* test. Data from multiple groups were analyzed by 1-way ANOVA, followed by Tukey’s multiple comparisons test. Statistical significance was determined as *P* < 0.05.

### Study approval.

Human studies were approved by the Northern Sydney Local Health District Human Research Ethics Committee. Written informed consent was received from participants prior to inclusion in the study. All animal experiments were performed in accordance with the National Health and Medical Research Council of Australia’s Code for the Care and Use of Animals for Scientific Purposes and were approved by the Northern Sydney Local Health District Animal Ethics Committee.

## Author contributions

QC conceived and designed the research, performed and interpreted the results of experiments, analyzed data, prepared figures, and drafted and revised the manuscript. CH provided some animal samples and revised the manuscript. HY and YS performed some animal experiments. AJG and AC performed histologic analysis. MF had input into experimental design and was responsible for providing the i-body. CGH, KKL, and CFT were responsible for biochemical characterization of the i-body. CAP and XMC conceived and designed the research, interpreted the results of experiments, analyzed data, and revised the manuscript. All authors approved the final version of the manuscript. CAP is the guarantor of this work and, as such, had full access to all the data in the study and takes responsibility for the integrity of the data and the accuracy of the data analysis.

## Supplementary Material

Supplemental data

## Version 1 01/11/2022

In-Press Preview

## Version 2 02/22/2022

Electronic publication
