# Supplementary material for: A single-domain i-body, AD-114, attenuates renal fibrosis through blockade of CXCR4
Source: JCI Insight. 2022 Feb 22;7(4):e143018. doi: 10.1172/jci.insight.143018 (PMC8876455; doi:10.1172/jci.insight.143018)
Supplement: Supplemental data [file jciinsight-7-143018-s016.pdf]

Supplemental data

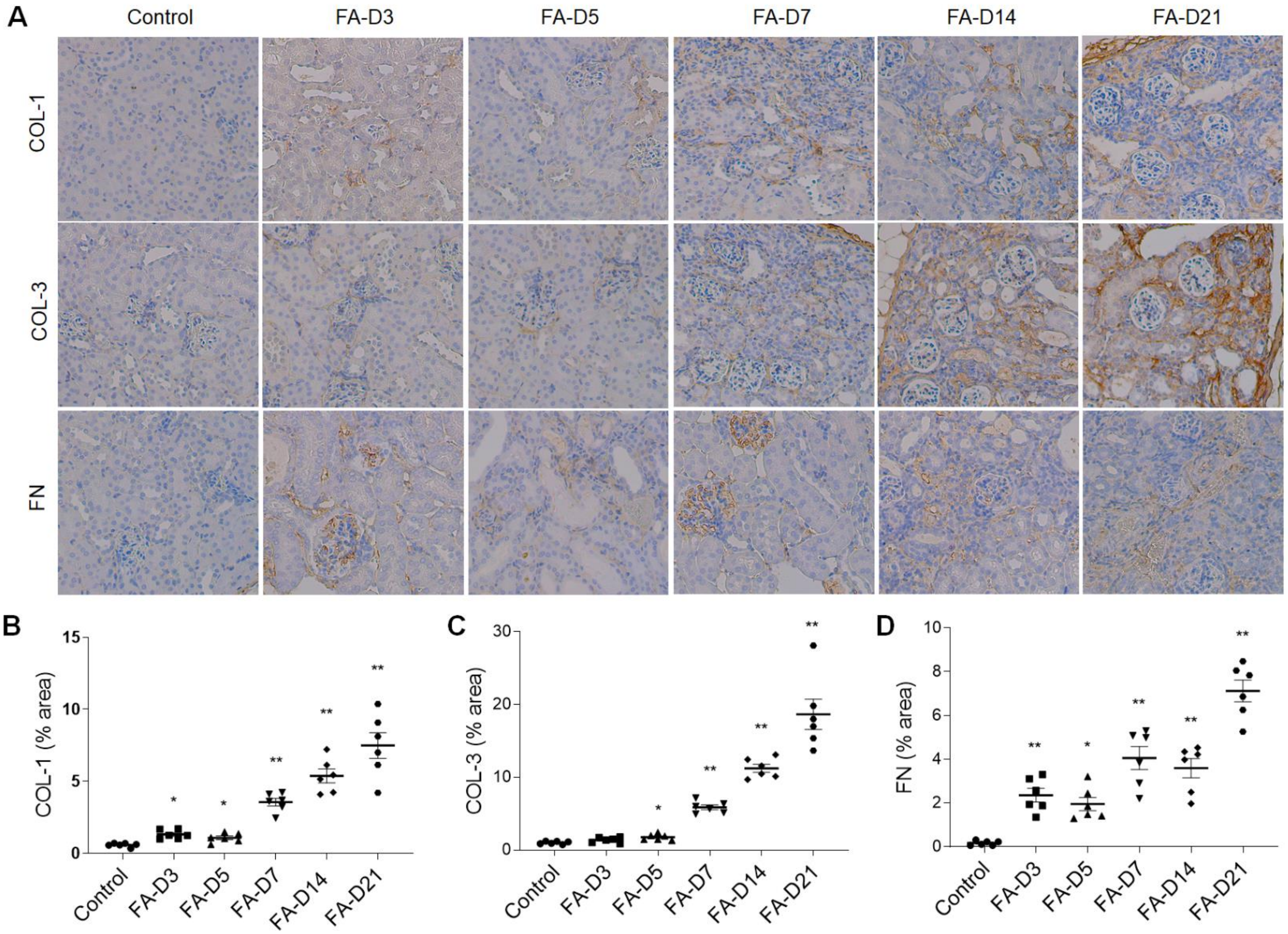

Supplemental Figure 1. IHC staining and quantitation of fibrotic markers COL-1, COL-3 and FN at different timepoints after FA injection in mice. Representative images were shown in (**A**). (**B-D**) Quantitation of COL-1, COL-3 and FN immunohistochemical staining. Original magnification: ×200 in all. Statistical analysis was performed using one-way ANOVA followed by Tukey's multiple comparisons test. Results are presented as mean±SEM. *P<0.05, **P<0.01. n=6.

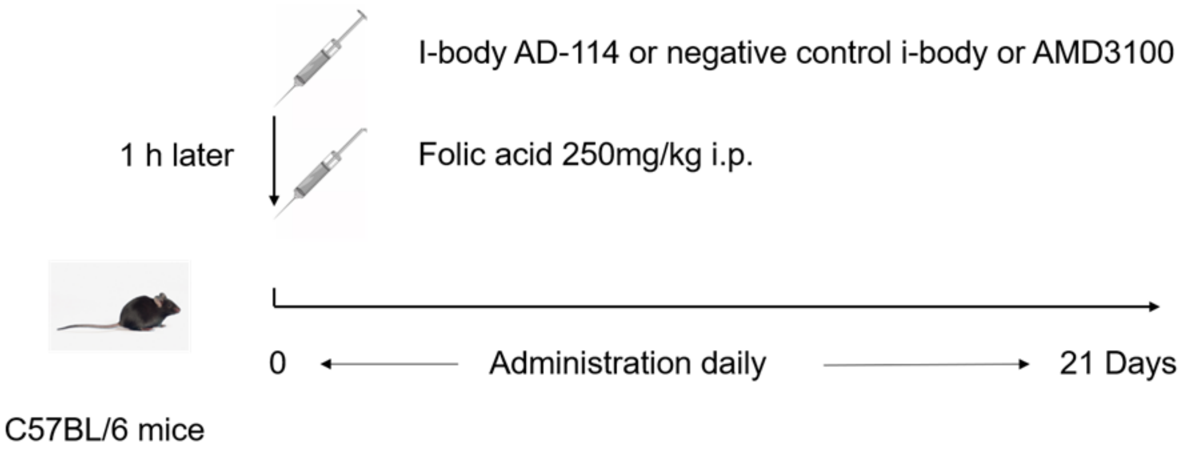

Supplemental Figure 2. Schematic representation of the timeline of FA and i-body administration in the preventative study. Male mice at 6-8 weeks of age were injected i.p. with FA to induce fibrosis. Mice were then randomly allocated to receive treatment of AD-114, negative control i-body or AMD3100 i.p. 1h prior to FA injection and then dosed daily until day 21.

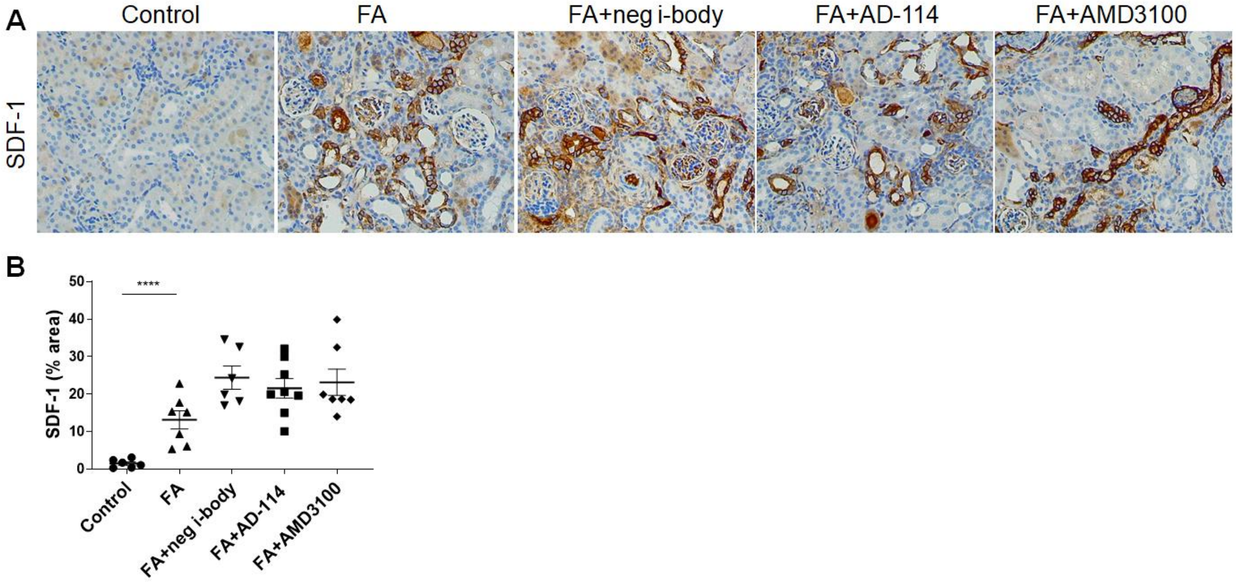

Supplemental Figure 3. AD-114 did not affect the SDF-1 expression. (**A**) Representative images and (**B**) quantitative analysis of SDF-1 immunohistochemical staining in kidneys of mice from Animal study 2 (preventative studies).

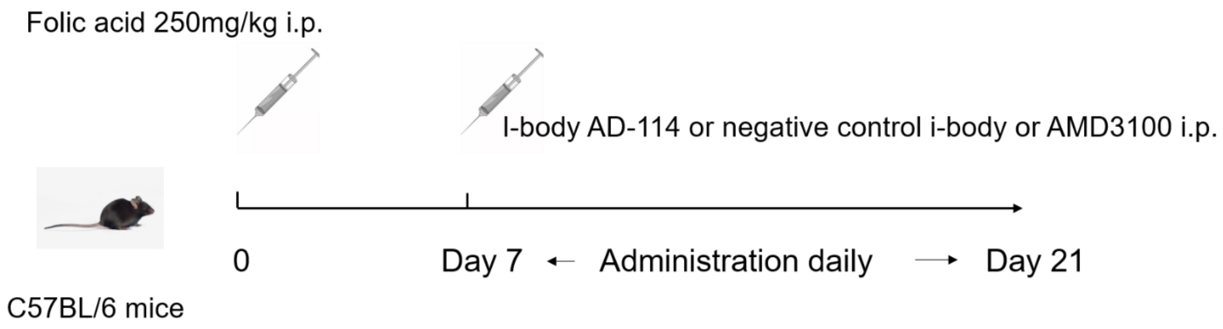

Supplemental Figure 4. Schematic representation of the timeline of FA and i-body administration in the therapeutic study. Male mice at 6-8 weeks of age were injected i.p. with FA to induce fibrosis. Mice were then randomly allocated to receive treatment of AD-114, negative control i-body or AMD3100 i.p. from day 7-21.

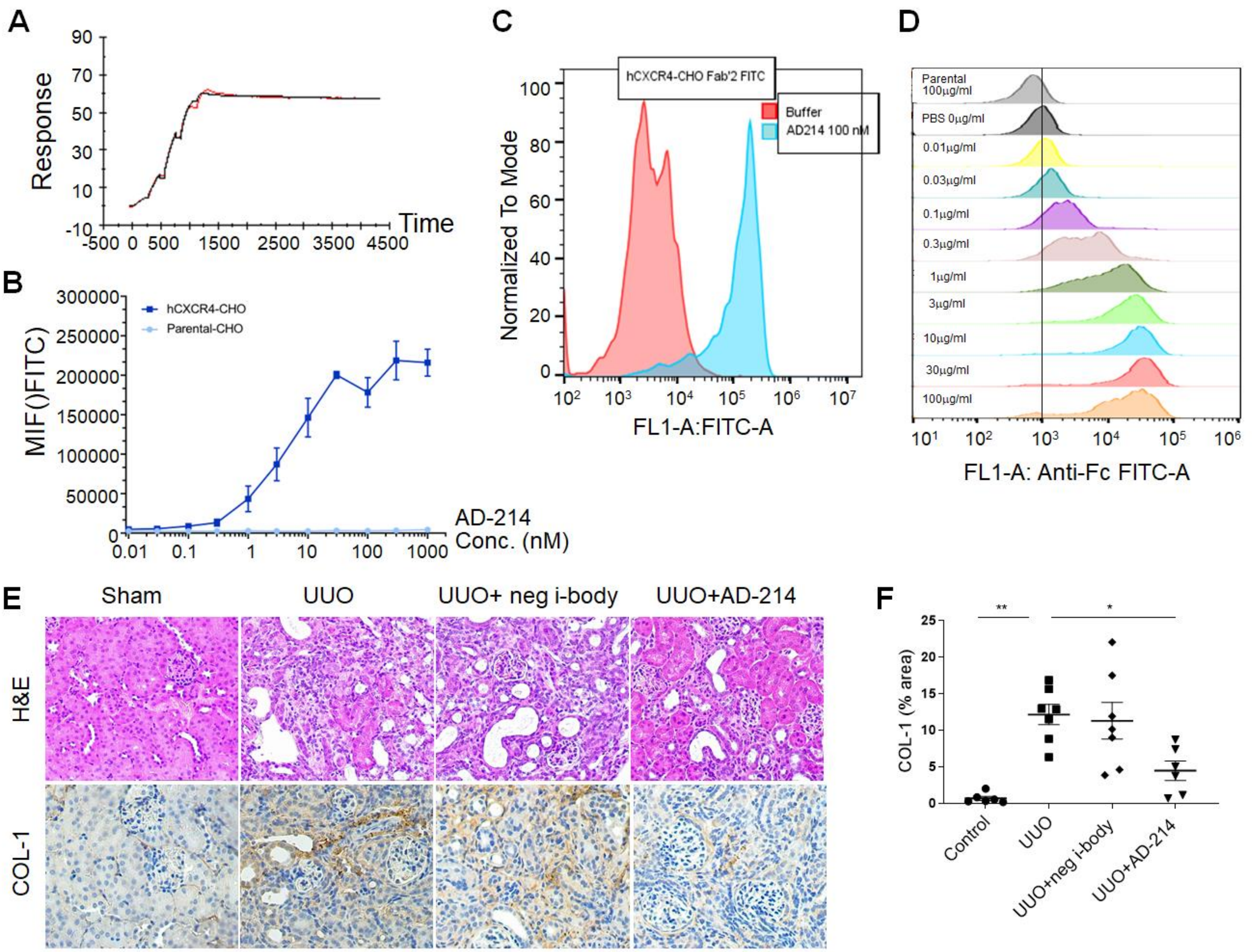

Supplemental Figure 5. Fc fused I-body AD-114 (AD-214) ameliorated UUO-induced renal fibrotic response. (A-D) AD-214 bound with high affinity to CXCR4. (**A**)AD-214 bound to human CXCR4 lipoparticles with affinity of ~4pM. (**B**)AD-214 binds to CXCR4 expressing CHO cells was detected using a fragment rabbit anti-Human IgG, Fcγ fragment specific F(ab')2. Data represents mean ± SEM of 3 replicates. (**C**) Bound AD-214 (100nM) to hCXCR4 expressed on CHO cells was detected using anti-human-Fc-Fab'2-FITC. (**D**) AD-214 can bind to CXCR4 expressed on human CD3+ T-cells. AD-214 was directly labelled with Alexa Fluor 647 (AD-214-647) and detected using flow cytometry. (**E-F**) Mice were dosed with negative i-body or AD-214 i.p. the following day of UUO and were then administrated every second day until day 14. (**E**) Representative images of H&E staining and IHC staining for COL-1. (**F**) Quantification of stained area as percentage of total area. Statistical analysis was performed using one-way ANOVA followed by Tukey's multiple comparisons test. Results are presented as mean±SEM. *P<0.05, **P<0.01. n=6-8. Original magnification: ×200.

Supplemental Table 1. The sequence of primers used for quantitative RT-PCR.

Species	Target	Forward (5'-3')	Reverse (5'-3')
Mouse	α-SMA	CATCTTTCATTGGGATGGAG	TTAGCATAGAGATCCTTCCTG
	β-Actin	CAGCTGAGAGGGAAATCGTG	CGTTGCCAATAGTGATGACC
	F4/80	CCTGGACGAATCCTGTGAAG	GGTGGGACCACAGAGAGTTG
	FSP-1	GGAGCTGCCTAGCTTCCTG	TCCTGGAAGTCAACTTCATTGTC
	IL-1β	TGTGAAATGCCACCTTTTGA	TGTCCTCATCCTGGAAGGTC
	IL-6	AAGAAATGATGGATGCTACC	GAGTTTCTGTATCTCTCTGAAG
	iNOS	GGGTCACAACTTTACAGGGAGT	CTCTCCACTGCCCCAGTTTT
	Msr1	AAAGGGAGAGAAGGGGAGTG	GCATGACACAGGAACCAATG
	Mrc1	CAAGGAAGGTTGGCATTTGT	CCTTTCAGTCCTTTGCAAGC
	TNF-α	CTGTAGCCCACGTCGTAGC	TTGAGATCCATGCCGTTG
	Vimentin	CCAACCTTTTCTTCCCTGAA	TGAGTGGGTGTCAACCAGAG
Human	COL-4A1	CGGGTACCCAGGACTCATAG	GGACCTGCTTCACCCTTTTC
	FN	GCGAGAGTGCCCCTACTACA	GTTGGTGAATCGCAGGTCA
	GAPDH	AGCCACATCGCTCAGACAC	GCCCAATACGACCAAATCC
	MMP-2	ATAACCTGGATGCCGTCGT	AGGCACCCTTGAAGAAGTAGC
